# Performance of Quad Mass Gyroscope in the Angular Rate Mode

**DOI:** 10.3390/mi12030266

**Published:** 2021-03-04

**Authors:** Sina Askari, Mohammad H. Asadian, Andrei M. Shkel

**Affiliations:** MicroSystems Laboratory, University of California, Irvine, CA 92697, USA; asadianm@uci.edu (M.H.A.); ashkel@uci.edu (A.M.S.)

**Keywords:** MEMS, gyroscope, angular rate, quad mass gyroscope, inertial sensor

## Abstract

In this paper, the characterization and analysis of a silicon micromachined Quad Mass Gyroscope (QMG) in the rate mode of operation are presented. We report on trade-offs between full-scale, linearity, and noise characteristics of QMGs with different Q-factors. Allan Deviation (ADEV) and Power Spectral Density (PSD) analysis methods were used to evaluate the performance results. The devices in this study were instrumented for the rate mode of operation, with the Open-Loop (OL) and Force-to-Rebalance (FRB) configurations of the sense mode. For each method of instrumentation, we presented constraints on selection of control parameters with respect to the Q-factor of the devices. For the high Q-factor device of over 2 million, and uncompensated frequency asymmetry of 60 mHz, we demonstrated bias instability of 0.095${}^{\circ }$/hr and Angle Random Walk (ARW) of 0.0107${}^{\circ }$/$\sqrt{\mathrm{hr}}$ in the OL mode of operation and bias instability of 0.065${}^{\circ }$/hr and ARW of 0.0058${}^{\circ }$/$\sqrt{\mathrm{hr}}$ in the FRB mode of operation. We concluded that in a realistic MEMS gyroscope with imperfections (nearly matched, but non-zero frequency asymmetry), a higher Q-factor would increase the frequency stability of the drive axis resulting in an improved noise performance, but has challenges in implementation of digital control loops.

## 1. Introduction

Microelectromechanical Systems (MEMS) gyroscopes have been employed successfully in many sensor applications [1], including roll-over detection for safe driving in the automotive industry [2,3], rotation rate measurement for high-end gaming in consumer electronics [4], human motion tracking in Virtual Reality (VR) and Augmented Reality (AR) applications [5], drilling guidance in oil or gas exploration [6], north finding [7], space applications [8], and navigation applications [9].

**Figure 1:**
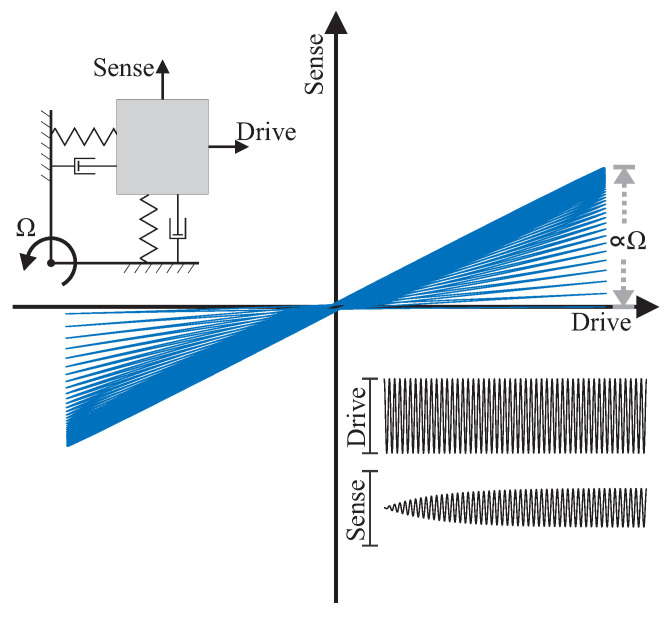
Response of a CVG operating in the angular rate mode. The response is due to rotation along the Z-axis, orthogonal to the page. Overlay plot of drive and sense axes oscillations illustrates a harmonic motion along the drive axis and transients along the sense axis, with the steady-state amplitude along the sense axis proportional to the applied angular rate.

**Figure 2:**
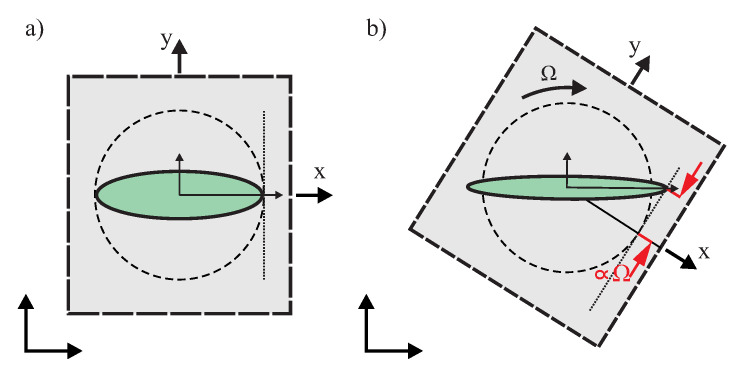
Theoretical response of a CVG to a rate of angular rotation Ω, in the gyroscope rotating frame: (**a**) no rotation, (**b**) applied a constant rotation.

MEMS Coriolis Vibratory Gyroscopes (CVGs) are based on transfer of energy between primary and secondary modes of the gyroscope due to the Coriolis force coupling, in response to an input rotation [10]. Figure 1 shows this exchange of energy between the drive and sense modes, when a device is experiencing a rotation. The drive axis is under continuous oscillation along the drive axis using a feedback loop for amplitude stabilization and the Coriolis acceleration induced motion is sensed along the orthogonal sense axis. A MEMS CVG can be configured to operate in the rate mode, to measure the angular rate of rotation, or in the whole-angle mode, to measure the absolute angle of rotation [11]. In the rate mode of operation, the resolution floor of the gyroscope is described by bias instability and Angle Random Walk (ARW), where ARW is a figure of merit to quantify the angle wander resulting from the integration of noise in the rate signal over time [12]. From the Mechanical-Thermal Noise (MTN) model, a noise-equivalent rate in an open-loop gyroscope, which defines a lower bound of the performance, the Quality-factor (Q-factor) of the sense mode, frequency mismatch between the drive and the sense modes, and the drive mode amplitude are the parameters influencing the performance of the angular rate gyroscopes, [13]:

(1) $$\begin{array}{c} \Omega_{rw}\approx \sqrt{\frac{k_{B}T\omega_{y}}{A^{2}M\omega_{x}^{2}Q_{y}}{\left[1+{\left(\frac{Q_{y}\left(\omega_{y}^{2}-\omega_{x}^{2}\right)}{\omega_{y}\omega_{x}}\right)}^{2}\right]}^{-1}}\;, \end{array}$$

where $\Omega_{rw}$ is the noise equivalent rotation rate, $k_{B}$ is the Boltzmann’s constant, *M* is the effective mass, *T* is the operating temperature measured in Kelvins, *A* is the drive axis amplitude, $\omega_{x}$ and $\omega_{y}$ are the drive and sense resonant frequencies measured in rad/s, and $Q_{y}$ is the sense-mode Q-factor. Equation (1) indicates, for example, the smaller the frequency mismatch ($\omega_{x}-\omega_{y}$) and the higher the Q-factor, the lower the characteristic noise of a CVG. Figure 2 illustrates schematically the oscillation deflection in the rotating coordinate frame. The output sensed along the Y-axis is proportional to the input angular rate, where MTN in (1) defines the minimum detectable signal. Thus, mode-matching [14] and the Q-factor maximization [15] are the key strategies to augment the measurement sensitivity and reduce the mechanical thermal noise at any operational frequency.

Dissipation mechanisms in MEMS resonators and CVGs have been studied and several structural designs have been implemented to achieve low noise characteristics. A dual mass gyroscope architecture, with decoupled tines reported by [16] and with synchronization lever mechanism by [15], demonstrated the Q-factor of 125,000. Analogous to the dual mass, a device with four masses and coupling frames has been introduced by [17] and the Quadruple Mass Gyroscope (QMG) with anti-phase lever mechanisms by [18], demonstrating the Q-factor of 450,000. In this implementation, the neighboring frames were in a coupled arrangement and moved in anti-phase relative to each other. A QMG with as-fabricated frequency mismatch of 0.2 Hz and the Q-factor of 1,170,000 was demonstrated with 0.88 ${}^{\circ }$/hr in-run bias instability and ARW of 0.06 ${}^{\circ }$/$\sqrt{\mathrm{hr}}$, operating in the Force-to-Rebalance (FRB) mode [19]. A two-mass Dual Foucault Pendulum (DFP) architecture, [20], was designed to provide a minimal realization of a mode-matched dynamically balanced lumped mass gyroscope. The DFP was believed to provide advantages over the QMG architecture while reducing complexity of the design. An epitaxially-encapsulated Dual Foucault Pendulum (DFP) operating at 15 kHz with the Q-factor of 1,150,000 demonstrated an in-run bias instability of 1.9 ${}^{\circ }$/hr and an ARW of 0.075 ${}^{\circ }$/$\sqrt{\mathrm{hr}}$ in the open-loop rate mode of operation [21]. Q-factors of over 9,290,000 were demonstrated, introducing a methodology for making the anchor losses observable by nulling the thermoelastic damping under specific cryogenic temperatures [22]. Other successful implementations of MEMS gyroscopes are Disk Resonator Gyroscope (DRG) and Bulk Acoustic Wave (BAW) disk gyroscope. These architectures are based on flexural vibrational modes. A silicon DRG with an active temperature compensation has been reported with the Q-factor on the order of 80,000 operating at 14 kHz with in-run bias instability of 0.012 ${}^{\circ }$/hr and ARW of 0.002 ${}^{\circ }$/$\sqrt{\mathrm{hr}}$ [23]. An epitaxially-encapsulated polysilicon DRG operating at 264 kHz with the Q-factor of 50,000 demonstrated an in-run bias instability of 3.26 ${}^{\circ }$/hr and ARW of 0.36 ${}^{\circ }$/$\sqrt{\mathrm{hr}}$, [24]. A BAW disk gyroscope was demonstrated in [25], with the Q-factor of up to 1,380,000 at 2.745 MHz center frequency, in an actively controlled vacuum chamber. In an ideal case, the modal symmetry and balanced motion of the sensing element in QMG, DRG, DFP, and BAW disk resonators were conceived to cancel out the reaction forces and moments acting at the anchor locations, thus mitigating the dissipation of energy through the substrate. In case of the DFP, for example, the anchor loss was demonstrated to be 9 times lower than the Thermoelastic Damping (TED) limit of the Q-factor [22].

The QMG device described herein has evolved considerably from the first introduction of the concept in [18], in terms of its structure and control architecture, demonstrating an excellent modal symmetry and exceptional Q-factor. Features of the design discussed here are categorized into three design iterations: (QMG-I) with mass symmetry and an external lever mechanism [18,26], (QMG-II) with mode-ordering, as well as an internal and external levering, comb-finger drive electrodes and parallel-plate sense electrodes [27,28], and, (QMG-III) with a complete symmetry of the design, including internal and external levering, with differential parallel-plate drive and sense electrodes [29,30], and vacuum sealing with getters. Table 1 summarizes the key parameters of these iterations, the performance numbers and the corresponding publications, a visual comparison of the layouts is provided in [31] Section 3.2.4. Out of the three iterations, QMG-II was operated in the closed-loop and the rest in the open-loop mechanizations. Therefore, we are comparing design versus performance parameters between the first two generations. From the latest design iteration, QMG-III, we evaluated Devices Under Test (DUT), summarized in Table 2, to illustrate the effect of different parameters of the devices to their performance characteristics.

In this work, we discuss the noise performance and the effect of vacuum sealing on QMGs with three different Q-factors, ranging from a 1000 (DUT1) to a 2,000,000 (DUT3), Table 2. DUT3 with the highest Q-factor was used to demonstrate capabilities of the QMG design and MEMS technology. The reported devices were instrumented to operate in the rate mode.

The material of this paper is organized as follows. In Section 2, we present a discussion on the structural design of QMG, using as an example DUT3. In this section, we also introduce the electrostatic scheme for actuation and detection of the orthogonal modal frequencies and present the initial frequency response characterization. We will also investigate the identification of the energy dissipation mechanisms in a QMG sensor. In Section 3, we report a procedure of electrostatic tuning of the frequency split, as well as analyze and discuss strategies for implementation of control algorithms and selection of control parameters for sensors with different Q-factors. The limitations of a high-Q mode-matched MEMS CVG in terms of the scale-factor nonlinearity and measurement bandwidth in the open-loop rate and force-to-rebalance rate modes are experimentally analyzed in Section 4. In Section 5, a discussion on noise performance analysis of DUTs in the open-loop rate mode is presented and compared to the FRB rate mode of operation. The same section discusses two methods for deriving ARW and bias instability from the Allan Deviation (ADEV) and the Power Spectral Density (PSD) analysis. Both methods identified and modeled random errors of the gyroscope output, where ADEV was extracted from the time-domain data and PSD was extracted from the frequency-domain data. Furthermore, finally, stability of the drive resonance frequency is characterized and correlated to noise performance of the device. Section 7 concludes this paper with summary and outlook.

## 2. Quad Mass Gyroscope (QMG)

**Figure 3:**
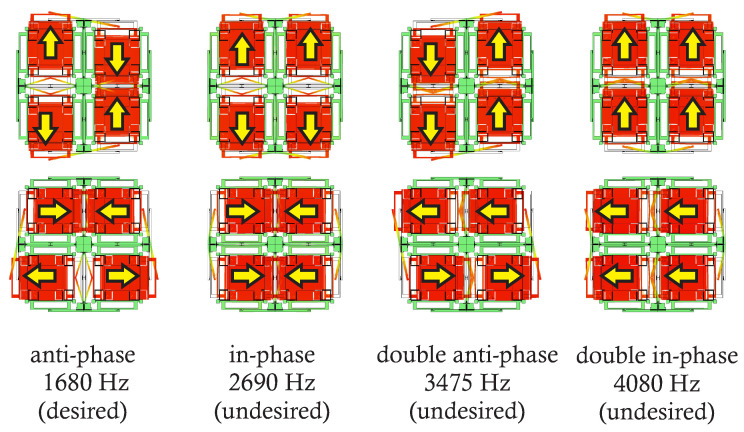
Eigen-frequency simulations of a Quad Mass Gyroscope (QMG), showing the four degenerate modes of vibration and the frequency of each resonance mode. The vibrational modes are ordered to place the desired anti-phase mode at the lowest frequency [31].

A QMG comprises four coupled identical oscillators, providing an X-Y symmetry of the resonant structure [34]. The coupled oscillators have four degenerate resonance modes: (1) anti-phase, (2) in-phase, (3) double anti-phase, and (4) double in-phase, Figure 3. The in-phase and double anti-phase modes are not independent and are coupled by the Coriolis force, thus they are not utilized for gyro operation in the QMG design. The anti-phase and double in-phase modes are independent and sensitive to the Coriolis coupling and can be used for gyro operation. However, the double in-phase mode is sensitive to external linear accelerations and should be avoided as the operational mode. The anti-phase motion of masses during the operation assures minimization of the total reaction forces and moments at the anchors, resulting in reduction of energy losses through the substrate, and therefore the preferential mode for gyro operation [35].

**Figure 4:**
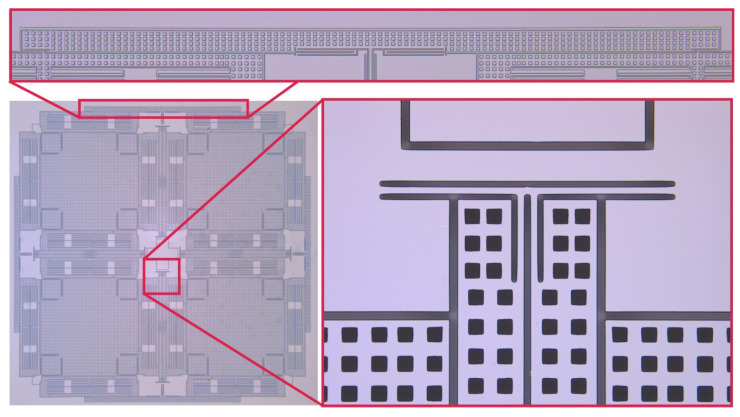
Four external lever mechanisms and four pairs of internal secondary beam resonators, responsible for ordering the eight vibrational modes of a QMG and for placing the desired anti-phase mode at the lowest frequency [36].

QMGs have one operational mode and three parasitic vibrational modes, which are sensitive to external linear and angular accelerations. In order to suppress sensitivity to environmental shock and vibrations and improve mechanical stability of the sensor’s structure, the order and frequency of vibrational modes were designed using suspension elements for mode-ordering, [29]. Four outer lever synchronization mechanisms and four pairs of inner secondary beam-coupling elements were incorporated in the suspension design to couple the proof-masses (see Figure 4). Advantages of these features of the design included widening of the frequency separation between desired anti-phase modes and parasitic in-phase modes, while shifting the in-phase modes to higher frequencies for common mode rejection of linear accelerations, decreasing the mode conversion losses and decreasing the drift induced by external vibrations.

While the anti-phase operation was intended to reduce the energy dissipation and improve the Q-factor along each orthogonal axis, the symmetric structure of the device provided a damping and stiffness symmetry, which was shown to improve the overall performance of the gyro operating in both the rate and the rate-integrating modes [18].

QMGs that were used in this study were fabricated using a Silicon-on-Insulator (SOI) process with a 100 μm device layer, 5 μm buried oxide layer, and a 500 μm handle wafer. In this design, the mass of each tine was ∼ $1.4\times 10^{-6}$ kg, the width of suspension beams was 10 μm, and the minimum trench width was 7 μm. The total footprint of the device was 8.6 mm ×  8.6 mm. The devices were diced and individual sensors were attached to ceramic Leadless Chip Carrier (LCC) packages using eutectic bonding [37].

**Figure 5:**
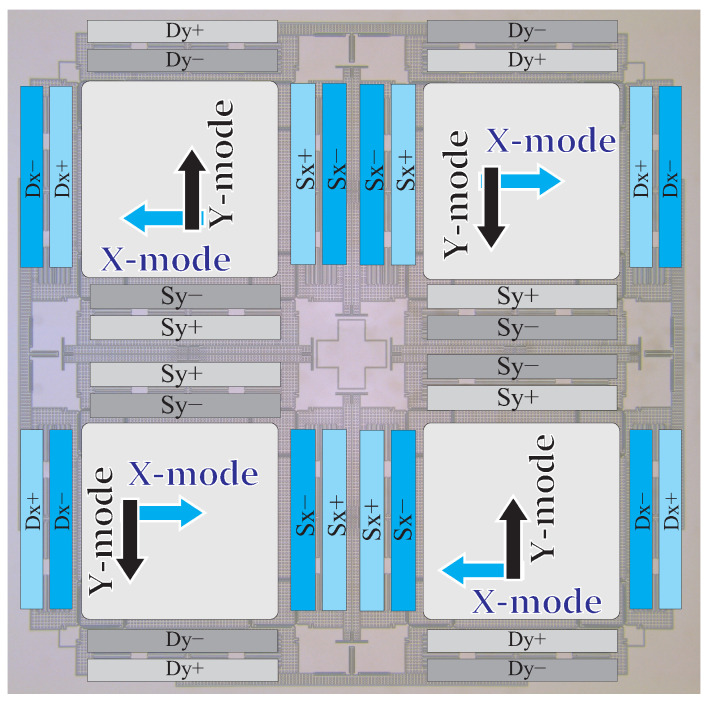
Layout of the QMG structure with symmetric features: identical proof-masses and identical drive and sense electrode structures overlaid with external forcers and internal pickoff electrodes, which are used to drive and sense each mode separately.

The sensors consisted of 16 pairs of differential parallel-plate electrode arrays with 7 μm capacitive gaps for excitation and detection of the drive and sense modes. Every four pairs of differential electrodes cover one proof-mass. In four-mass symmetric configuration, differential electrodes for drive are located on the outer side of the structure, and differential electrodes for sense are located on the inner side of the structure. Altogether, the parallel-plates formed 3.7 pF active capacitance between the proof-mass and the differential electrodes for each X- and Y-modes. The differential drive signals were applied to all four masses symmetrically. For example, along the X-axis for the bottom two masses, the in-phase drive signal (+) was applied to the outer most electrodes and the out-of-phase drive signal (−) was applied to the inner electrodes; this configuration is reversed for the top two masses. The differential sense signals from all masses were lumped to one pickoff signal of the detection circuit. Figure 5 shows the arrangement of electrodes for excitation and detection of the anti-phase mode of motion. The differential pairs of electrodes were labeled (“+” and “−”), for both the drive (Dx and Dy) and the sense (Sx and Sy) electrodes. These electrodes were wirebonded, such that they summed under the same subset (e.g., the Dx+ signal arrives in the LCC package to 4 different pads and distributes to 4 electrodes).

### 2.1. Frequency Response Characterization

**Figure 6:**
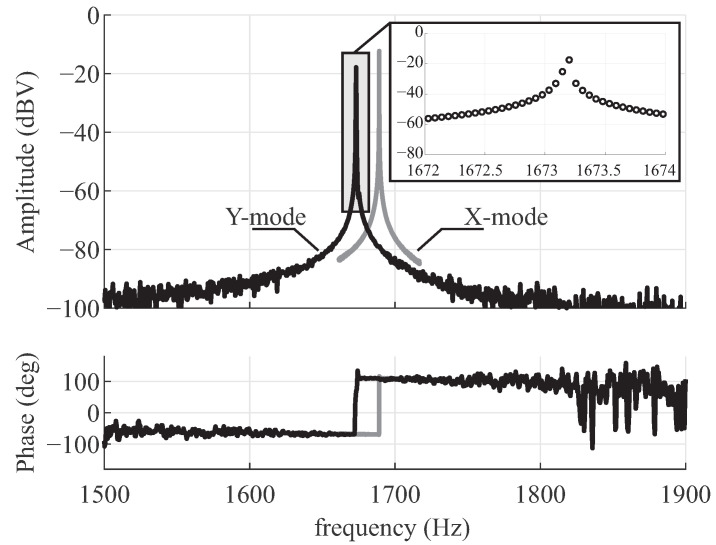
Experimental results of the anti-phase drive (X-mode) and the sense (Y-mode) frequency responses. Illustrating the increase in amplitude spectral density near the resonance frequency of the modes.

**Figure 7:**
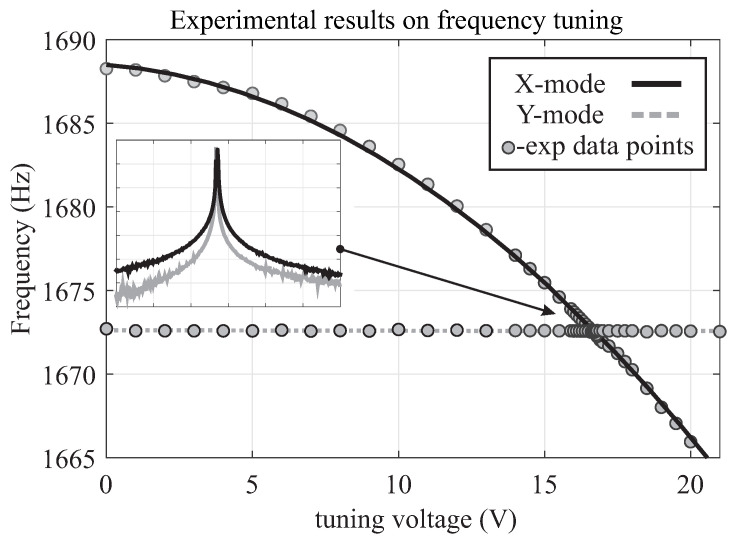
Experimentally obtained frequency response of the QMG showing the frequency separation changes between the orthogonal axes by applying DC bias to the X-mode pickoff electrodes. Illustrating the X-mode resonance frequency decreases with increase tuning voltage while Y-mode resonance frequency keeps unchanged.

The initial frequency response characterizations were carried out using a custom analog signal conditioning printed circuit board, utilizing a charge amplifier (AD8034 Op Amps) with a feedback resistor ( 1 -MΩ Vishay resistor), for capacitive detection. An HF2LI lock-in amplifier from Zurich Instruments was used for the experiments. An Electromechanical Amplitude Modulation (EAM) scheme was utilized to remove parasitic feedthrough from the forcer to pickoff electrodes [38]. A  100 kHz carrier signal was applied to the proof-masses and balanced by DC biases on all drive electrodes (equal DC voltages were applied to all differential drive pair electrodes, Dx and Dy). An AC drive signal generated by the network analyzer was applied to the drive electrodes, a differential pair Dx for the X-axis or a differential pair Dy for the Y-axis. The amplitude of the pickoff signal was estimated after demodulation at the carrier frequency and the drive frequency. The phase of the delayed carrier was initially set to 0, while the amplitude of the pickoff signal was monitored. A slightly delayed carrier was used for demodulation, allowing for an optimal phase setting of the EAM. The frequency response along the X-axis and Y-axis are plotted in Figure 6, demonstrating an anti-phase resonant frequency at 1673 Hz and an as-fabricated frequency mismatch of 15 Hz. The frequency mismatch was electrostatically tuned down to 60 mHz ( 36 ppm) using 16.58 Volts DC bias applied to the sense electrodes along the X-axis, with the resolution of 3.5 digits of the power supply, Figure 7.

### 2.2. Q-Factor Measurement

Using the same setup as described in Section 2.1, the Q-factor was estimated by measuring the ringdown time. The ringdown time, τ, is defined as the time that it takes for the settled drive amplitude to drop down to 1/e of the initial drive amplitude under free vibration [39], and is measured in seconds. This parameter is used to extract the Q-factor, Q=πfτ, where *f* is the resonant frequency measured in Hertz.

In MEMS resonators and CVGs, the Q-factor is a figure of merit and a measure of the overall damping from all possible loss mechanisms in a system. The primary energy dissipation mechanisms in MEMS resonant structures are viscous damping, Thermoelastic Damping (TED), anchor loss, surface-related losses, and electrical damping [40]. The overall Q-factor is the reciprocal sum of Q-factors from different loss mechanisms and is limited by the dominant loss mechanism in the system:

(2) $$Q_{Total}^{-1}=Q_{Viscous}^{-1}+Q_{TED}^{-1}+Q_{Anchor}^{-1}+Q_{Others}^{-1}.$$

**Figure 8:**
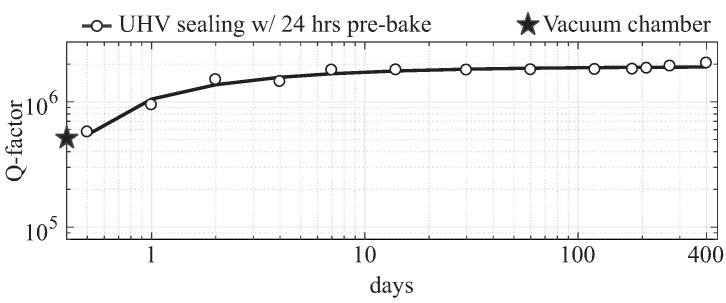
The Q-factor measurements over a long period of time revealed vacuum stability inside a sealed cavity after vacuum sealing (DUT3). The improvement of the Q-factor is related to the pumping of residual gas molecules by the activated getter [37].

In order to suppress the effect of viscous damping, the DUTs were sealed using an Ultra-High Vacuum (UHV) sealing process [37]. For vacuum sealing of sensors, LCC packages were pre-baked at 400 ${}^{\circ }$C in a vacuum furnace for 7 h in high vacuum (<10 μTorr) prior to the die attachment. A vacuum compatible eutectic alloy composed of 80% gold and 20% tin (AuSn 80/20) was used for the die attachment. QMG sensors were sealed in vacuum at <0.1 mTorr using the SST 3150 sealing furnace. The Q-factors above 1 million were repeatedly achieved on QMGs using the developed sealing process. Details of the vacuum sealing process were reported in [37]. The long-term vacuum stability was characterized for over 1 year for DUT3 and demonstrated that the Q-factor does not degrade over time, and even exhibits a continues improvement, Figure 8. The long-term ultra-high vacuum condition was enabled by surface desorption prior to sealing, pumping of residual gases by passive getters, and defect-free solder reflow in the sealing area.

**Figure 9:**
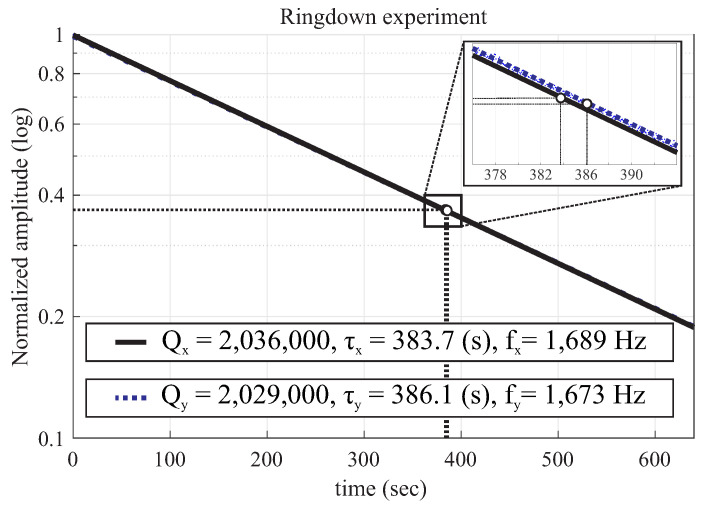
Ringdown time measurements revealed the Q-factor as high as 2 million, on both X-axis and Y-axis, after UHV sealing.

For the DUT3 in this example, the X-mode Q-factor was measured at $Q_{x}=$ 2,036,000, which corresponds to $\tau_{x}=$ 383.7 (s) at $f_{x}$ = 1689 Hz, and along the Y-mode Q-factor was measured at $Q_{y}=$ 2,029,000, which corresponds to $\tau_{y}$ = 386.1 (s) at $f_{y}$ = 1673 Hz, Figure 9. The inverse of time decay mismatch between both modes $\Delta \left(1/\tau \right)=\left(1/\tau_{x}-1/\tau_{y}\right)$ was estimated to be $1.1\times 10^{-4}$ Hz. The Q-factors after vacuum sealing approached the $Q_{TED}$ limit of the structure, which was modeled using finite element simulation to be at 3.5 million [41].

The discrepancy between the measured Q and the simulated TED for DUT3 was due to other dissipative mechanisms contributing to the overall Q-factor. The second dissipation mechanism in the highest-Q DUT3 is the anchor loss. In an ideal case, QMG provides fully balanced linear and angular momentum with zero net force and moments on the anchors, when the device is operating in the anti-phase mode. However, the fabrication non-idealities would break the symmetry. From the measured Q factor and estimated TED limit, the $Q_{anchor}$ is expected to be higher than 4.5 million. The anchor loss limit can be experimentally evaluated in a cryogenic chamber at ∼100 Kelvin where the TED limit is eliminated. The procedure is explained in [22,42]. However, due to the limited number of probes in our cryogenic probe station setup, the anti-phase mode of QMG could not be excited fully differentially, hence it was not possible to accurately estimate the anchor loss contribution.

## 3. Performance Analysis

In this section, the statistical analysis of the Zero Rate Output (ZRO) of three QMG sensors with different Q-factors and different levels of symmetry, but similar operational frequencies, are discussed. The purpose of this analysis is to provide insight into factors contributing to lower noise performance by identifying device-specific error parameters, and subsequently analyze the effect on control algorithms, and relate to complexity of control algorithm implementations. The variation in the Q-factors of DUTs is attributed to the different vacuum sealing conditions. DUT 1 and DUT 2 were packaged without getter with a pre-bake duration of 4 h and 12 h, respectively. DUT 3 was packaged with a pre-bake duration of 24 h and getter activation. Using the Q-factor measurements at different pressures in a vacuum chamber prior to the vacuum sealing, the residual cavity pressure for DUT 1, 2, and 3 were estimated to be ∼1 Torr, ∼40 mTorr, and <100 Torr, respectively. Table 2 summarizes parameters of the selected sensors. The residual frequency mismatch after electrostatic tuning, control accuracy, frequency stability, and the induced noise on the ZRO output are discussed next.

### 3.1. Frequency Split (Δf) Extraction

**Figure 10:**
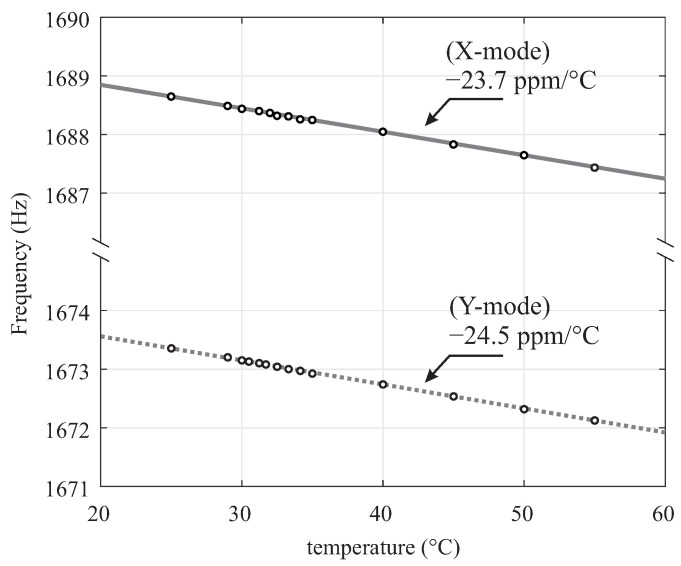
Experimental results of the TCF along X-axis and Y-axis of the sensor with as-fabricated Δf of 15 Hz. The measurement was performed in a thermally-controlled environment, with temperature ranging from 25 to 55 ${}^{\circ }C$, and temperature fluctuations within 0.04 ${}^{\circ }C$ at each measurement point.

Fabrication imperfections in QMGs are the primary causes of frequency mismatch between the drive and sense modes. The lowest possible noise floor is achieved when the frequency mismatch is small [43]. To correct for residual imperfections, an electrostatic frequency tuning was used, which is discussed in Section 2.1. The frequency split/mismatch (Δf) was estimated at each discrete DC voltage level from the resonant frequency along the X-axis and the Y-axis. The extraction of Δf for high Q-factor sensors under the tuned condition (typically <1 Hz) is challenging. The temperature dependency of frequency sweep characterization was also investigated. As an example, for a typical room temperature fluctuation of 2 ${}^{\circ }C$ during the frequency sweep characterization, the resonant frequencies would shift 80 mHz. To illustrate this, we characterized experimentally the Temperature Coefficient of Frequency (TCF) of the sensor. Figure 10 demonstrates the measured TCF of the sensor in a thermally-controlled environment with an average TCF value of $-24\;\mathrm{ppm}{/}^{\circ }C$.

**Figure 11:**
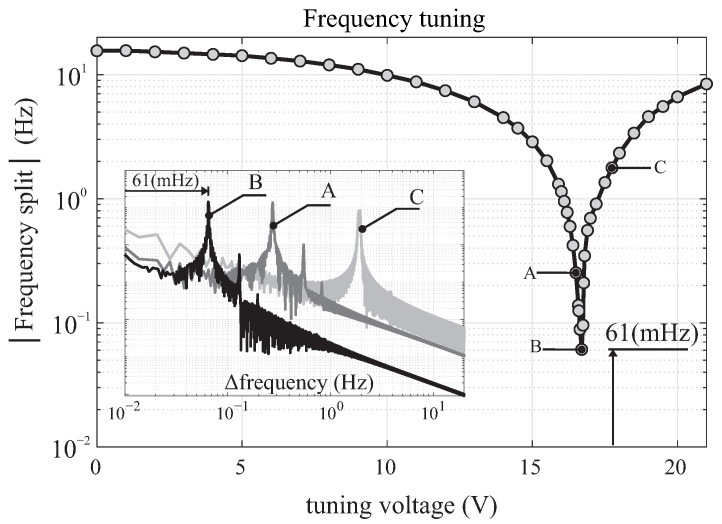
Estimation of |Δf| by monitoring the peak in the power spectrum of the nearly-matched region for a high Q-factor device. Inset figure shows the PSD of the drive signal at the corresponding reference points. Results are experimental.

In the nearly-matched frequencies, the Δf was extracted by FFT spectrum analyzer of oscillations along the drive axis. Figure 11 shows an extracted Δf using the procedure, confirming the ability to reach an optimal tuning voltage value at 16.15 V, achieving Δf < 1 Hz. The inset plot illustrates examples of PSD with three DC voltage levels (A, B, C) at 15.80, 16.15 and 17.35 V, with an estimated Δf of 250, 60 and 1900 mHz, respectively. The described method enables a real-time observation of the Δf for high Q-factor devices, while actively adjusting the applied DC tuning voltage.

**Figure 12:**
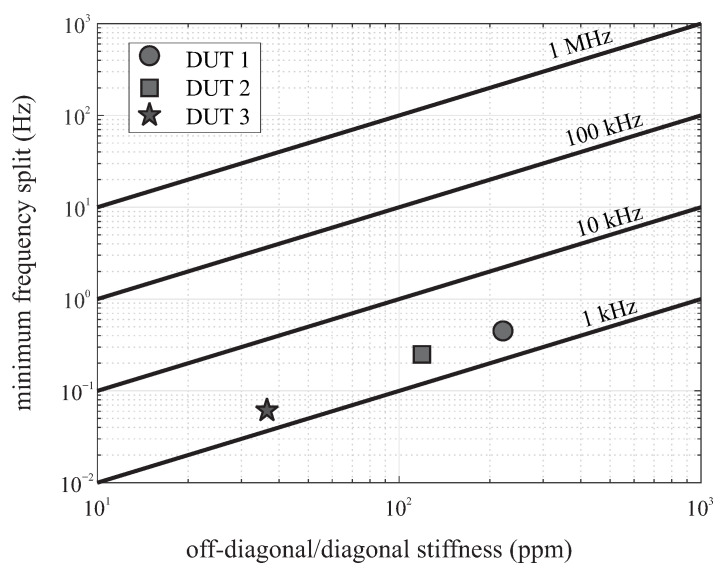
Minimum of frequency split (Δf) between drive and sense modes as a function of off-diagonal stiffness. Straight lines represent different drive oscillation resonant frequencies.

The variation in the width of spring elements across a die due to fabrication imperfections results in a misalignment between the orientation of the principal axis of elasticity and the orientation of electrostatic forces along the drive and sense axes defined by the layout. Due to asymmetry and misalignment of axes, non-zero off-diagonal components appear in the stiffness matrix. The achievable tuning accuracy of frequency mismatch depends on the off-diagonal stiffness and the nominal frequency of operation. Compensation of off-diagonal stiffness using electrostatic spring softening requires tuning electrodes along 45 degree orientation with respect to drive and sense. Figure 12 illustrated the frequency mismatch accuracy versus ratio of off-diagonal to diagonal stiffness in part per million for different resonant frequencies. The level of imperfection in DUTs were not identical since different processes were used for their fabrication.

### 3.2. CVG Control Algorithm

**Figure 13:**
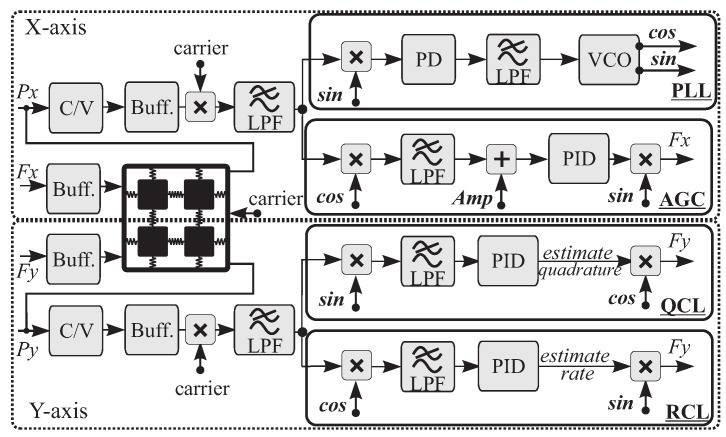
Control structure for operating devices in the rate mode. AGC and PLL were activated along the drive axis of the device (X-mode). QCL and RCL were activated along the sense axis of the device (Y-mode).

The CVG control algorithm was implemented based on the IEEE Std 1431 [44]. The digital control of the characterization was carried out using the HF2LI lock-in amplifier. Four primary control loops were implemented for the rate mode characterization, including Phase-Locked Loop (PLL), Amplitude Gain Control (AGC), Quadrature Control Loop (QCL), and Rate Control Loop (RCL), Figure 13. Each loop comprises: (1) a demodulator for demodulating a received signal from the device, either along X-axis or Y-axis, into in-phase (*cos*) or in-quadrature (*sin*) signals, (2) a low pass filter (LPF) for passing only low-frequency component, (3) a PID controller with a set point for controlling the DC component of the demodulation, and (4) a modulator for modulating the controlled signal to a higher frequency defined by the local reference oscillator signal. The PLL loop has two extra components, which are Phase Detector (PD) and Voltage-Controlled Oscillator (VCO). Using these control loops, a gyro can be configured to operate in the open-loop rate mode under the following conditions: (a) PLL only, (b) PLL and AGC, (c) PLL, AGC, and QCL, or (d) closed-loop rate mode where all four loops (PLL, AGC, QCL, and RCL) are established, also known as closed-loop FRB rate mode.

The PLL generates a reference frequency by tracking the resonant frequency of the drive mode, which is done by a VCO with negative feedback to a phase detector. The phase detector compares the phase of the received signal with respect to the local oscillator and generates an error signal; the PLL is called “locked” when the error signal is zero. The AGC loop maximizes the in-phase component of the drive signal to a pre-defined set point value, and the QCL loop nulls the in-quadrature component of the sense signal. The RCL loop is used to estimate the input rate from the in-phase component of the sense signal in the open-loop configuration, or from the voltage applied to null the rate signal in the FRB rate mode.

### 3.3. Control Accuracy

In this section, we highlight challenges in stabilization of the control loops for devices with different levels of symmetry and the quality factor, and derive the corresponding hardware requirements. A PID controller is needed to track and stabilize the amplitude in the drive direction, phase of the drive oscillation frequency, amplitude of the quadrature and rate parameters in the sense direction from demodulated components of the drive and sense signals (also known as slowly-varying parameters). The general PID equation can be represented by:

(3) $$\begin{array}{c} u=K_{P}e+K_{I}\int e+K_{D}\dot{e}\;, \end{array}$$

where *u* is the control signal, *e* is the control error, and the controller parameters are $K_{P}$ (proportional), $K_{I}$ (integral) and $K_{D}$ (derivative) gains of the linear control architecture. In this work, only the PI controller was used for feedback to stabilize parameters (amplitude, phase, quadrature, and rate feedback). Each controller has a set point, bandwidth, and sampling rate. Experimental results revealed that the PI parameters for a low Q-factor device (high loop bandwidth) would fail to control the high Q-factor devices (low loop bandwidth), and vice versa. Applying PI parameters, which were selected for a high Q-factor device, to a low Q-factor device would result in a slower response, which is unfavorable for fast frequency tracking. Consequently, the loop bandwidth has to be set based on the device parameters. These parameters for the three selected devices (DUT1-DUT3) were set as follows: the −3 dB cutoff frequency of the loop filter in the PLL was selected to be around 100 Hz, centered at the resonant frequency of the drive axis. A phase shift occurred between the driving mass forcer signal (denoted by Fx in the diagram shown in Figure 13) and the phase detector in the PLL loop (marked as PID block in the diagram shown in Figure 13). The signal path includes a preamplifier buffer circuit, the device, a charge amplifier, a front-end buffer circuit, a carrier demodulation circuit, and a LPF. The phase setpoint of the PLL aligns this phase shift of the feedback signal with the forcer signal. The transfer function of a resonator PLL system dynamics is [45]:

(4) $$\Phi \left(s\right)=\frac{1}{\left(t_{c}s+1\right)},$$

where Φ is the resonator’s phase and $t_{c}=2Q/\left(2\pi f\right)$ is the exponential time constant. The output propagates to a low-pass filter, where the controller adjusts the phase Φ. The PI parameters of the PLL unit are responsible for the fast lock of the resonance frequency to the reference local oscillator. The high Q-factor resonators require a narrower bandwidth ($BW=1/t_{c}$), on the order of Q inverse, due to the transfer function characteristics of the system, whereas in low Q-factor resonators the bandwidth is much higher, again on the order of Q inverse.

For the DUTs in this study, the setpoints for the rate and quadrature loops were selected to be 0 Vrms and for the drive amplitude were selected to be 0.42 Vrms, to utilize the full-scale resolution from the amplifier output to the Analog-to-Digital Converter (ADC) with an input range of ±1.2 Volts.

**Figure 14:**
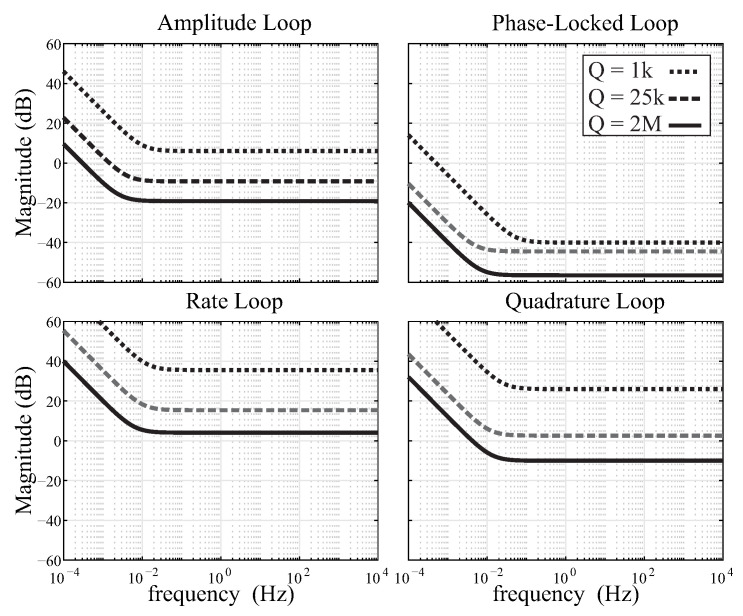
Illustrated configurations of PI parameters on the amplitude (AGC), phase (PLL), quadrature (QCL) and rate (RCL) loops for QMG devices with Q-factors ranging from 1000 to 2,000,000. PI parameters were scaled proportionally to the Q-factor of the device. Results are experimental.

To achieve the target bandwidth and stable loop conditions, different sets of PI parameters (units for $K_{P}$ are [V/Vrms] or [Hz/deg], and for $K_{I}$ are [V/Vrms/s] or [Hz/deg/s]) were selected and verified experimentally for devices with different Q-factors. For a Coriolis vibratory gyroscope in a self-oscillation mode (or using an external signal generator from PLL), the Routh-Hurwitz criterion to satisfy the stability is [46]:

(5) $$\begin{array}{cc} \; & K_{P}>\frac{m_{x}2\pi f_{x}}{G_{p}G_{f}Q_{x}},\\ \; & \omega_{c}K_{P}>K_{I}, \end{array}$$

where $G_{p}$ and $G_{f}$ are the gain buffer after pickoff and generated forcer signals, and $\omega_{c}$ is the cutoff frequency parameter of the LPF of the loop. The $G_{p}$ parameter was fixed across the three DUTs, but $G_{f}$ was adjusted accordingly and was selected to be lower when the Q-factor was higher. Thus, $K_{P}$ is inversely proportional to the Q-factor and the $G_{f}$. Given the above parameters and initial settings for each loop, $K_{P}$ and $K_{I}$ were implemented and results are shown in Figure 14. The figure illustrates the sensitivity constraints on the magnitude of the four primary CVG control loops vs. frequency. In the closed-loop configuration, the integrator’s coefficient $K_{I}$ stabilizes the proportional controller and zeros the steady-state error. As expected, the crossover integrator frequency (closed-loop bandwidth) component decreased as the device’s resonance Q-factor increased. Overall, the PI parameters of the control loop need to be adjusted based on the Q-factor of the device, affecting the speed of the control loops. The sampling rate of the input signals to the four primary CVG control loops (PLL, AGC, QCL, and RCL) were set to be at 130 kHz (between 5 and 10× of the device’s resonance frequency). In our experiments, the sampling rate of the PI digital controller run one order of magnitude faster than the crossover frequency, ensuring that any changes in the signal can be controlled.

### 3.4. Open-Loop Operation in the Rate Mode

In the open-loop CVG rate mode of operation, the drive mode was excited to a fixed amplitude $A_{0}$, with the use of AGC, and at the frequency $f_{x}$, and with the use of PLL. Due to the Coriolis coupling, the input rate causes the excitation of the sense mode channel, and the sense mode amplitude is proportional to the input rate.

**Figure 15:**
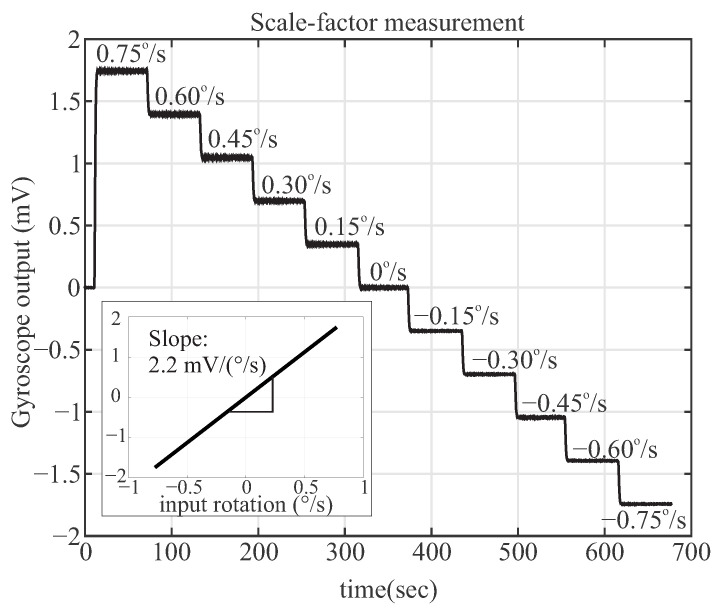
Characterization of angular rate response to clockwise and counter-clockwise rotation with different step-input amplitudes of 0, ±0.15, ±0.30, ±0.45, ±0.60 and ±0.75 ${}^{\circ }/s$, revealing an open-loop scale-factor of 2.2 mV/(${}^{\circ }$/s).

The scale-factor was extracted by applying a reference rotation using a rate table, with incremental step inputs of 0.25 ${}^{\circ }$/s in the clockwise and counter-clockwise directions. The open-loop scale-factor of 2.2 mV/(${}^{\circ }$/s) was obtained for the high Q-factor device. Figure 15 illustrates the device response over time for a small input rotation range, where the inset plot shows linearity of the input-output of the same dataset.

### 3.5. Force-to-Rebalance Operation in the Rate Mode

Similar to the open-loop, in the closed-loop CVG the drive mode was excited to a fixed amplitude $A_{0}$, with the use of AGC, and at the frequency $f_{x}$, with the use of PLL. An additional force was applied along the sense mode to null the response. This force is required to null the sense mode amplitude and is proportional to the input rate, thus the architecture is called Force-to-Rebalance (FRB) or closed-loop instrumentation of CVG operating in the rate mode.

The scale-factor extraction is similar to the described open-loop architecture, and, similarly, the ZRO data could be used for noise analysis. Due to inconsistencies in the sampling time interval during data recording of the Digital-to-Analog Converter (DAC) components of the hardware setup, the noise performance of the gyroscope’s output rate in the FRB mode was estimated from the input to the RCL loop (open-loop rate estimate) ADC component multiplied by inverse of the loop gain, rather than the output of DAC component (Fy signal, shown in Figure 13). However for the scale-factor, the output was estimated from DAC component from maximum and minimum fluctuation response, even though with sampling time inconsistency. The FRB rate voltage output was then converted to an equivalent rotation rate in ${}^{\circ }/s$.

## 4. Scale-Factor Nonlinearity and Bandwidth

The advantage of high-Q MEMS CVGs operating in the mode-matched condition is a high sensitivity to the input rotation and its ability to measure low angular rates. However, the linearity of the scale-factor [47] and the measurement bandwidth [48] are limited when the sensor is operating in the open-loop rate mode. The scale-factor linearity and the bandwidth limit of the sensors operating in the open-loop and closed-loop modes were characterized experimentally.

**Figure 16:**
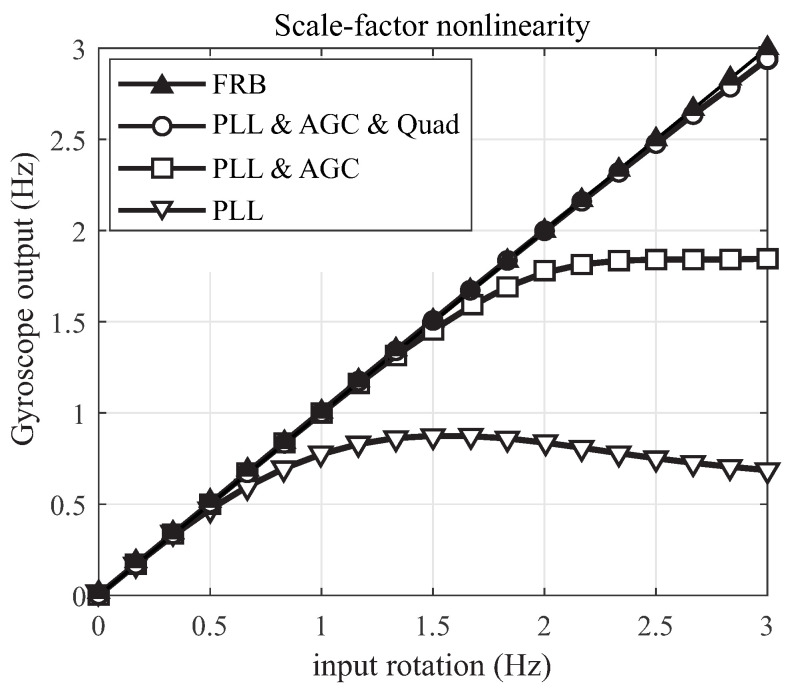
Experimental measurements of the scale-factor nonlinearity in DUT1, operating in the mode-matched condition with different configurations of control loops.

For linearity analysis, an input rotation in the range of angular rates from 0 ${}^{\circ }$/s to 1080 ${}^{\circ }$/s with an increment of 60 ${}^{\circ }$/s was applied using an Ideal Aerosmith 1571 rate table. The linearity of the input-normalized output under different loop configurations were compared experimentally only for the DUT1 operating in the mode-matched condition, Figure 16. DUT1 with low Q-factor was selected for flexibility of adjusting parameters and repeating the experiment at several input angular rotations. The results demonstrated that the linear range of operation is limited in the open-loop operational mode, that is when only the drive axis control loop PLL was established. The linear range increases when both PLL and AGC were activated. An extended scale-factor linearity was observed when devices operated in the closed-loop sense configuration (FRB). Based on these results, the FRB fully compensates for nonlinearity in the output response for the input rotation range from 0 to 3 Hz. It is expected to see a similar trend for DUT2 and DUT3 by activating individual control loops, however with a smaller linear region due to quality factor of these samples.

**Figure 17:**
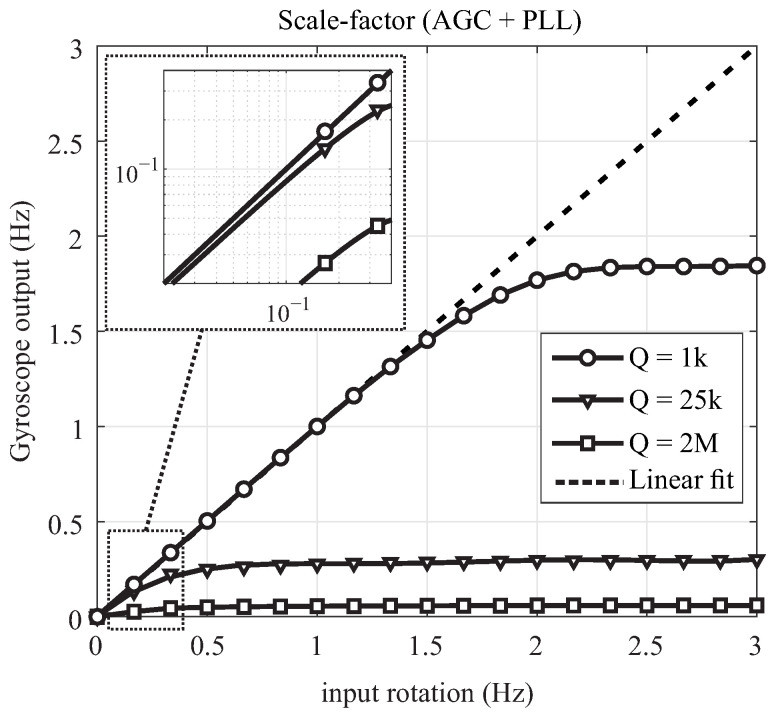
Experimental results of the scale-factor nonlinearity in sensors with different Q-factors and mode-matched condition, operating in the open-loop rate mode (PLL and AGC loops are enabled).

Next, we repeated measurements in the open-loop configuration, when control loops (PLL and AGC) were activated across all three DUTs. Figure 17 demonstrates the scale-factor nonlinearity in the open-loop rate mode for devices with different Q-factors, operating in the mode-matched (or nearly-matched) condition. As expected, the linear input-output range of operation becomes narrower as the Q-factor increases, confirming the sensitivity of resonator’s bandwidth in the open-loop operational mode relative to its drive frequency over Q-factor, $f_{x}/Q$.

**Figure 18:**
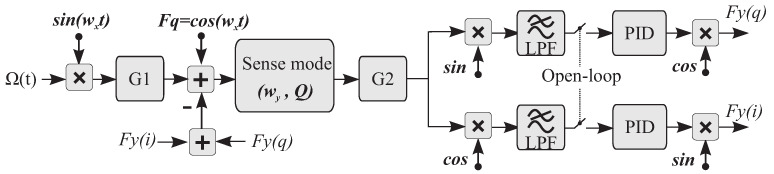
Simplified block diagram of the open-loop and closed-loop rate QMG MEMS gyroscope.

In a mode-matched device, the bandwidth is determined by the Q-factor of the device, whereas for a mode mismatched device the BW is dominated by the Δf [49]. Generally, a higher bandwidth can be achieved by increasing the damping coefficient (lowering the Q-factor) or operating in the mode-mismatched condition (increasing the frequency mismatch Δf), [50]. We support this observation by a diagram shown in Figure 18. In the diagram, the parameters for the drive amplitude (A) and the mass (m) are grouped as G1 = –2 mAΩ, and the circuit and buffer gains are grouped as G2. The notations $F_{y}\left(i\right)$ and $F_{y}\left(q\right)$ represent the in-phase and in-quadrature forces applied along the sense axis of the device. The input drive voltage F(t) along the sense axis in the open-loop case is

(6) $$F\left(t\right)=-2mA\Omega sin\left(w_{x}t\right)+F_{q}cos\left(w_{x}t\right),$$

and in the closed-loop (FRB) case is

(7) $$F\left(t\right)=-2mA\Omega sin\left(w_{x}t\right)+F_{q}cos\left(w_{x}t\right)+F_{y}\left(q\right)+F_{y}\left(i\right).$$

The Laplace transform of each of the shown components are as follows:

(8) $$\begin{array}{cc} Sense(s) & =\frac{1/M}{s^{2}+\left(w_{y}/Q\right)s+w_{y}^{2}},\\ LPF(s) & =\frac{w_{c}}{s+w_{c}},\\ PI(s) & =K_{P}\left(1+\frac{K_{I}}{K_{P}}s\right),\\ OL(s) & =Sense(s)LPF(s)F(s),\\ FRB(s) & =(OL(s)PI(s))/(1+OL(s)PI(s)). \end{array}$$

**Figure 19:**
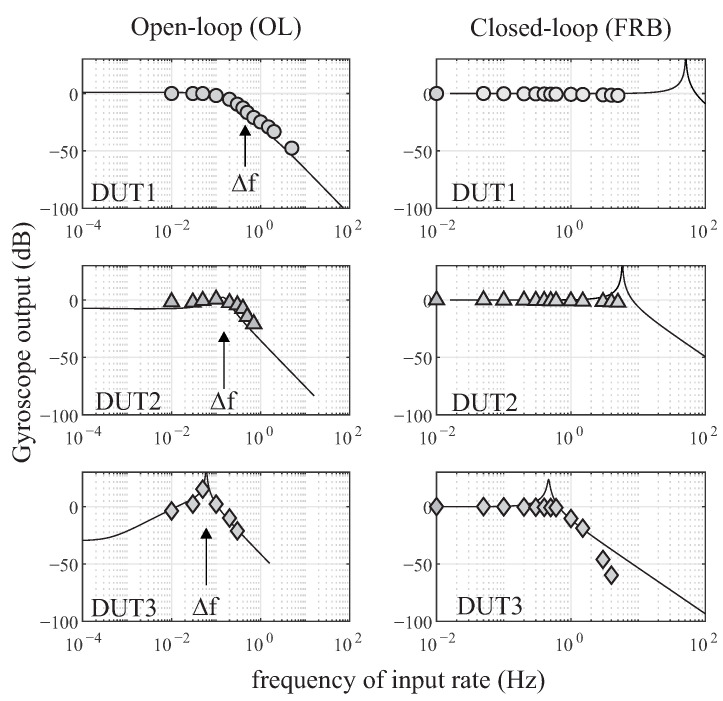
Simulation of the gyroscope bandwidth overlaid with experimental measurement points, operating in the open-loop (**first column**) and closed-loop (**second column**). Three devices with different Q-factors were used and each row represents one device.

To filter out the excitation amplitude of the sense mode resonance, the selection of the LPF cutoff frequency $w_{c}$ is typically 3 times lower than the Δf. The selection of the PI controller gains $K_{P}$ and $K_{I}$ are also device dependent and were discussed in Section 3.3. The frequency analysis of the open-loop OL(s) and the closed-loop FRB(s) of the gyroscope model with respect to the input rotation Ω(s) was performed in the Matlab environment, and shown in Figure 19. The analysis was repeated on all three DUTs with different Q-factors. For DUT1 and DUT2 with low and medium Q-factors, the open-loop BW analysis shows a strong dependency to the frequency split, but for the DUT3 with a high Q-factor of 2 M and Δf of 60 mHz, the –3 dB was dominated by the Q-factor of the device and estimated to be 0.5 mHz, which is two orders of magnitude lower than the frequency split. The BW limit observed in the open-loop operation can be compensated if the device was operated in the FRB mode. Simulation of parameters of the three DUTs shows that a higher BW is possible when FRB is selected as a preferable mode of operation. This conclusion was also verified experimentally and shown in the graph of Figure 19. However, this should be understood that the FRB amplitude range is limited to the DAC forcer amplitude resolution.

The BW of the QMG sensors were experimentally derived. Sinusoidal stimulus commands with varied frequencies, from 0.01 Hz to 5 Hz, were applied to the rate table. The experiment was repeated for the three sensors in the open-loop and the FRB rate mode mechanizations, Figure 19. From the rate output measurement of each mode, the –3 dB range resolution was extracted to be at 100, 227 and 790 mHz, which is in a close agreement with the tuned Δf of the QMG devices under electrostatic tuning conditions of 450, 250 and 60 mHz, respectively. Operating the device in the FRB configuration resulted in a higher bandwidth, independent from the frequency split of the device. This was confirmed on DUT1 and DUT2. However, for the high Q-factor sensor (DUT3), and for the input angular rate of rotation around 1 Hz, the FRB utilized the full amplitude range available of the hardware along the sense axis of the device. Therefore, there was no enough force authority to fully null the input rate above this limit, and the forcer signal along the sense axis (Fy) was saturated, resulting in a faulty loop operation. This constraint resulted in –3 dB loss at 1 Hz, Figure 19.

## 5. Noise Analysis

The breakdown of all noise processes in the ZRO output of a gyroscope can be described by:

(9) $$\sigma_{T}^{2}\left(\tau \right)=\sigma_{ARW}^{2}\left(\tau \right)+\sigma_{B}^{2}\left(\tau \right)+\sigma_{QN}^{2}\left(\tau \right)+\sigma_{RRW}^{2}\left(\tau \right)+...\;,$$

where ARW, *B*, QN and RRW represent the Angle Random Walk, Bias Instability, Quantization Noise, and Rate Random Walk, respectively, and the corresponding Allan variance $\sigma^{2}$ at any given τ averaging time. To extract these noise parameters individually, two statistical methods were used and compared, one in the time domain and another in the frequency domain. We analyzed the amplitude fluctuations and signal power over frequency of the rate output. Furthermore, the frequency stability was also analyzed to identify the noise sources. These methods are discussed next.

### 5.1. Allan Deviation (ADEV)

**Figure 20:**
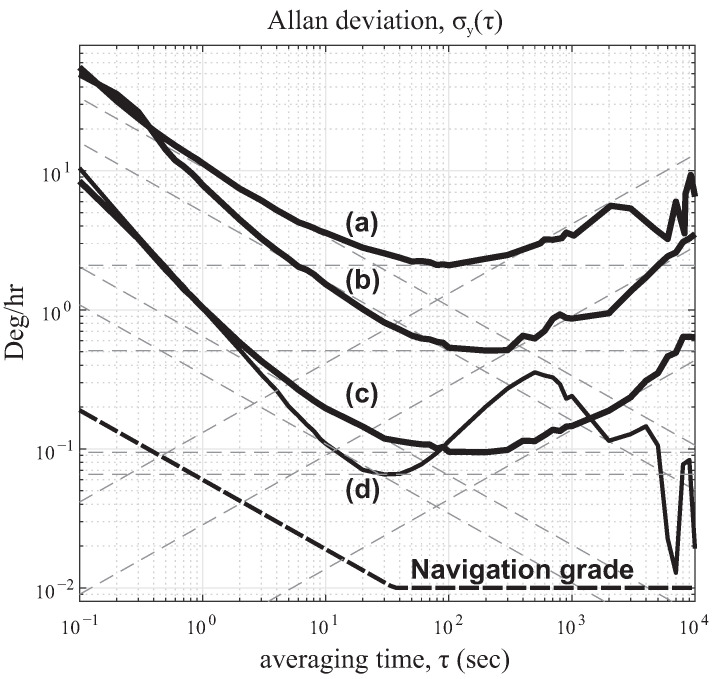
Noise characteristics of QMG with different Q-factor conditions with curve fit lines estimating ARW (slope –1/2), bias instability (slope 0) and RRW (slope +1/2) for QMG in the open-loop operation for (**a**) Q = 1k, (**b**) Q = 25k, (**c**) Q = 2M, and (**d**) in the closed-loop (FRB) operation mode, Q = 2M.

To characterize the short-term stability of the gyroscope, a two-sample deviation was measured over different time intervals. When the device was operated in the rate mode, the higher the Q-factor of the sense mode and the lower the frequency split between the drive- and the sense-modes, the lower ARW and a higher signal-to-noise ratio of the gyro response. In a still condition (no input rotation was applied), data was recorded for 16 h with the sampling rate of 10 Hz in the form of in-phase and in-quadrature data samples. The recording length was enough to provide an estimated ARW with 0.2% error from the ADEV plot, bias, and RRW. The data collection for the ZRO experiment was conducted in a lab environment without any thermal compensation. Frequency mismatch of <450 mHz was achieved under electrostatic tuning for the three DUTs, summarized in Table 3. The initial gyroscope bias (offset) across all DUTs were also extracted. In the open-loop mechanization, only PLL and AGC were activated. The bias instability of 0.09 ${}^{\circ }$/hr and the ARW of 0.01 ${}^{\circ }$/$\sqrt{\mathrm{hr}}$ were measured for the high Q-factor device (DUT3), Figure 20-line(c). As expected, the scale-factor of the sensor improved as the Q-factor increased. For the high-Q device, the noise characterization of the FRB mode of detection showed an improvement in the bias by a ratio of 0.69 and an improvement in the ARW by a ratio of 0.54. Compared to the open-loop result, in the FRB configuration it is likely the effect of frequency imperfection in the ZRO condition was eliminated [51], or despite the initial frequency mismatch condition of 60 mHz, in the FRB configuration an over-run operation might unintentionally tuned the frequency mismatch slightly as a result of the forcer being applied along the sense axis. All together, this likely led to a lower ARW. Therefore, the need for continuous tracking of frequency mismatch was identified as crucial in the open-loop and closed-loop operations.

### 5.2. Power Spectral Density (PSD)

**Figure 21:**
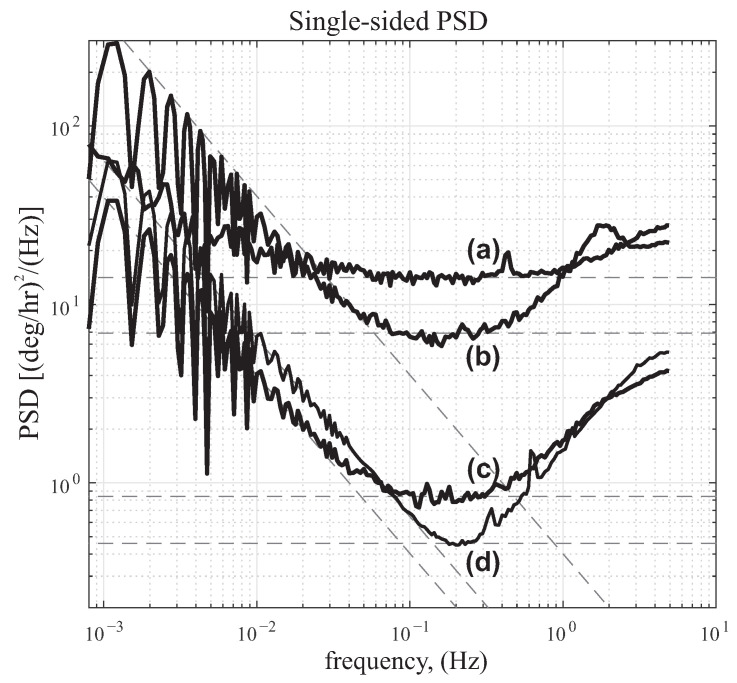
Rate PSD of static QMG data with curve fit lines estimating ARW (slope 0) and bias (slope –1). Lines (**a**–**d**) represent the same dataset and labelled in the time-domain analysis of Figure 20. Data sample rate is 10 Hz.

To verify the accuracy of statistical modeling derived from the time averaging Allan Variance method, the same data was processed using the logarithmic frequency averaging, also known as Power Spectral Density (PSD). The numerical processing procedure of the rate PSD can be found in [52]. This analysis essentially provides a single-sided PSD profile by averaging adjacent frequency bins. In the PSD plot, the bias instability is associated with 1/f noise and occurs at a slope of −1. The angle random walk is characterized by white noise of the rate output. It is where the flat part of the characteristic occurs, that is a frequency interdependent part of the plot (slope 0). Figure 21 shows the log-log plot of the PSD analysis of the QMG datasets, labeled as before. The fitted dashed lines represent the slope of −1 and 0. The PSD reproduced the estimated noise parameters of ARW, summarized in Table 3.

In the PSD analysis, the ARW component white noise is typically dominated and can be estimated well with a fit line (slope 0), whereas in the ADEV analysis the bias is dominated and estimated well with a line fitting (slope 0). In PSD, the estimation accuracy was reduced in the plot for low-frequency bins. In contrast, in ADEV, the uncertainty increased for long averaging time intervals.

Figure 21 shows this analysis and for a high Q-factor device it revealed the ARW of 0.008 ${}^{\circ }$/$\sqrt{\mathrm{hr}}$ and the bias of 0.0459 ${}^{\circ }$/hr. However, using the time domain integration on the same dataset, the bias instability was estimated to be 0.0946 ${}^{\circ }$/hr, the ARW to be 0.0107 ${}^{\circ }$/$\sqrt{\mathrm{hr}}$, and the RRW to be 0.0043 ${}^{\circ }$/hr/$\sqrt{\mathrm{hr}}$.

Both described methods are offline and require post-processing of the stored data. To demonstrate the effectiveness of both methods, the high Q-factor data was segmented into durations of 0–10 min, 0–20 min, 0–30 min, etc., all the way to 0–16 h, and then the ADEV and the PSD analysis were repeated. Both methods showed that the confidence of the estimated stability parameters improved as more data points were fed to the analysis. The high Q-factor data shows that the optimal size for bias estimation would reach 10% of its final value in 10 h for the ADEV method (Table 3), and from the PSD method in 7 h. Similarly, for the ARW estimation, 10% of its final value was reached in 3.5 h using the ADEV method, and in 5 h using the PSD method.

### 5.3. Frequency Stability

The stability of the drive mode oscillation at the resonance is a critical parameter as it directly relates to the scale factor of the device. Frequency and phase are related to each other by 2π, meaning that any instantaneous frequency changes (δf) in the drive mode are wrapped by 2π to maintain the phase changes (δΦ). For an oscillator, the instability can be approximated by δf/f≈−δΦ/2Q, where δf is a fluctuation in the drive oscillator frequency and δΦ represents changes in the phase [53]. Stability of the oscillator frequency is proportional to the Q-factor. The variation in oscillations along the drive axis was analyzed experimentally. The fluctuation in frequency or phase resulted in a phase error in the drive mode of oscillation, therefore a high Q-factor is desirable to minimize this error.

In a simplified analysis, the CVG can be viewed as a two-dimensional oscillator, where in the rate mode of operation, the output of the device is estimated from demodulation of the sense axis with respect to the frequency of the drive mode, as defined by the PLL. In the PLL, the phase is locked to the resonance center frequency in order to provide a reference frequency signal. Therefore, variations within this frequency or phase can directly translate to the phase error. Although it is outside the scope of this paper, it should be noted that a mode-mismatched operation would be an alternative method for device operation and would have its benefits, including less sensitivity to variations in demodulation phase. The Allan deviation analysis on frequency stability (δf/f) provides an estimate of the frequency noise processes. For inertial sensors, the frequency error is typically dominated by the frequency white noise [54]. Since the relation between the frequency and the phase is integral, as a result of integration, any small variation in frequency white noise (slope –1/2) resulted in phase error (slope +1/2). Therefore induced drift in white noise frequency contributes linearly to the RRW characteristics (slope +1/2) of the ZRO.

**Figure 22:**
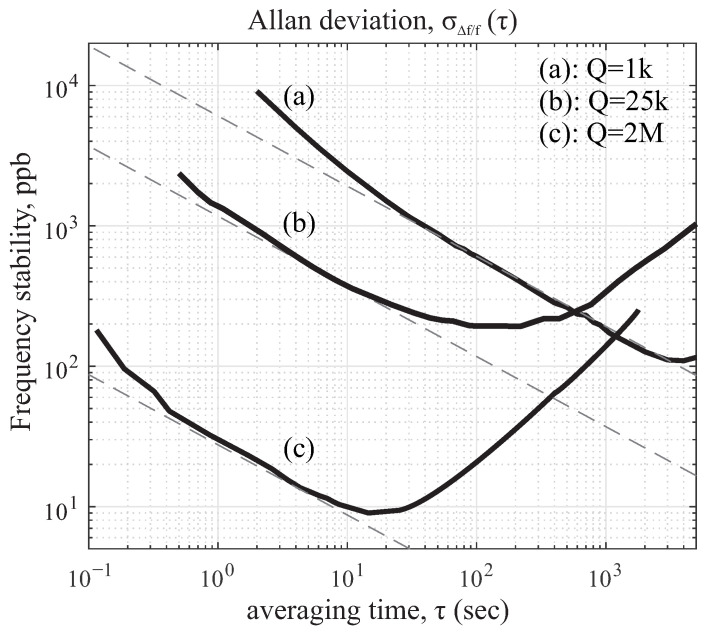
Characterization of the drive mode resonance frequency instabilities for three different Q-factors. The frequency white noise improved as the Q-factor increased.

Figure 22 shows the frequency stability analysis of the QMG devices with different Q-factors under the same room temperature environment, where the rate table operates in an enclosed thermal chamber. The frequency white noise was estimated to be 27.5, 1352.6 and 6072.5 ppb/$\sqrt{\mathrm{Hz}}$, which is in a strong agreement with the experimentally obtained RRW in Table 3. As expected, higher frequency stability was observed for devices with high Q-factors. Thus, supporting the prediction that maximizing the Q-factor helps to reduce environmentally induced noises in RRW (long term drift), including temperature and other long-term variations in the drive oscillator.

## 6. Discussion

The bias instability and ARW were optimized experimentally for the three DUTs and the highest values were reported. The drive amplitude and electrostatic frequency mismatch compensation were adjusted in an iterative process for each individual DUT to achieve the best possible noise performance. Maximizing the Q-factor (>2 M) and reducing the drive-sense frequency separation (<40 ppm) in QMG were demonstrated to improve the mechanical sensitivity and the noise performance by a factor of 25 and 10, respectively.

The reported performance in the open-loop makes QMG structure a viable design candidate to achieve navigation grade performance in silicon MEMS gyroscopes. However, we believe it might not be practical to operate the high-Q QMG in the open-loop angular rate mode of operation due to its limited bandwidth and scale factor linearity. Hence, the Force-to-Rebalance (FRB) is possibly a preferable mode of operation. It was demonstrated that in the FRB mode, in addition to bandwidth and linearity improvement, the rate random walk drifts less over time due to control of quadrature leakage into the Zero Rate Output (ZRO).

Additionally, in practical gyroscope applications, a device is expected to maintain its performance in a wide temperature range, where the resonant frequency of the drive and sense axes are subjected to change with temperature. This would result in shifting the drive-sense frequency separation, and would cause shift in sensitivity and demodulation phase. In a QMG architecture, the four fold symmetry allows robustness to the temperature variation since both drive and sense axes would expand identically when subjected to uniform temperature change. However, the fabrication imperfections break the structural symmetry and cause slight variations in the drive and sense frequency separation over temperature. This effect is significant when a high-Q gyro is operated in the (nearly) mode-matched open-loop mode. The robustness against temperature variations requires operation in the closed-loop control with an implementation of self-calibration. We identified the need for continuous monitoring of frequency mismatch. As in the open-loop, the frequency of the drive mode can be monitored through the PLL, but the sense mode frequency cannot be conveniently accessed. One can use mode reversal (intervals of switching operation) in FRB mode to extract and monitor frequency mismatches.

## 7. Conclusions

We presented the performance analysis of CVG devices, designed to operate in the rate mode in the nearly mode matched configuration. The paper discussed the corresponding control challenges involved. A highly symmetric device demonstrating the Q-factor of above 2 million was compared to 1000 and 25,000 Q-factor devices of the same design. The frequency split of all devices were electrostatically compensated to the lowest possible level given the configuration of the electrodes in layout of the DUTs. We demonstrated a possibility of achieving 0.09 ${}^{\circ }$/hr bias instability and a 0.01 ${}^{\circ }$/$\sqrt{\mathrm{hr}}$ ARW in the rate mode of operation in lab conditions (temperature fluctuations from 23.4 ${}^{\circ }C$ to 25.6 ${}^{\circ }C$), with no thermal compensations on the device level.

We described the structure of the CVG control algorithm and highlighted the hardware requirements for implementation. The criteria to achieve stable control loop conditions were examined on CVGs with different Q-factors. The dependence of the scale-factor nonlinearity on control loops was investigated in different combinations (PLL only, PLL+AGC, PLL+AGC+QCL, and FRB), which resulted in a linear full-scale dynamic range in the FRB mode. The tradeoff between bandwidth and sensitivity was investigated and shown experimentally on a CVG with different Q-factors operating in the open-loop rate mode. We verified and demonstrated that the frequency mismatch defines the operational bandwidth of the CVG in the open-loop mode, where the highest sensitivity is demonstrated for a lower Δf of mode mismatches. When the device was operated in the FRB mode, we observed deviations from linearity in the bandwidth analysis for devices with different Q-factors. We demonstrated that a higher Q-factor resulted in higher frequency stability, thus in lower rate random walk. These outcomes were predicted by our analytic analysis and supported in this paper experimentally.

We showed that a higher Q-factor and a lower frequency split can lead to the noise performance improvement by >100 fold in ARW, bias, and RRW. We derived the noise characteristic parameters, using both time domain and frequency domain analyses. Performing analyses on the same dataset showed that the ADEV method leads to representation of data with significantly lower noise characteristics compared to the PSD method, and should be considered as a lower bound on the noise performance. Regardless of the Q-factor, uncertainty in the noise parameters were lower on the flat portion (slopes 0, Figure 20 and Figure 21), that corresponded to bias instability in ADEV and ARW in PSD.

## Figures and Tables

**Table 1:** Progression of QMG performance characteristics.

Iteration	freq. [Hz]	Q-Factor	Δf [Hz]	ARW [${}^{\circ }/\sqrt{\mathrm{hr}}$]	Ref.
QMG-I	2177	1.17M	0.2	0.06	[19]
QMG-II	3047	980	0.15	0.02–0.05	[32]
QMG-III	2085	1.1M	0.2	0.04	[33]

**Table 2:** Characteristics of the three sensors used for the noise performance analysis.

Device ID	Q-Factor *	Drive Frequency [Hz]	Δf [Hz] (as-Fabricated)
DUT1	1050	2040	4
DUT2	25,750	2100	25
DUT3	2,036,000	1673	15

* The difference is due to different packaging conditions.

**Table 3:** Summary of device parameters and noise characteristics.

Device ID		DUT1	DUT2	DUT3	DUT3
Mode of Operation		OL	OL	OL	FRB
Quality factor (Q-factor)		1k	25k	2M	2M
Δf (tuned) [Hz]		450 m	250 m	60 m	60 m
SF [V/(${}^{\circ }$/s)]		85.7 μ	790 μ	2.2 m	2.6 m
offset [${}^{\circ }$/hr]		3.0542	1.0873	1.9497	-
ARW [${}^{\circ }$/$\sqrt{\mathrm{hr}}$]	ADEV	0.1770	0.0843	0.0107	0.0058
	PSD	0.1177	0.0576	0.0080	0.0038
Bias Instability [${}^{\circ }$/hr]	ADEV	2.0907	0.5096	0.0946	0.0655
	PSD	-	0.3938	0.0459	0.0647
RRW [${}^{\circ }$/hr/$\sqrt{\mathrm{hr}}$]	ADEV	0.1256	0.0300	0.0043	0.0107

## Data Availability

The data presented in this study are available on request from the corresponding author.

## Abbreviations

The following abbreviations are used in this manuscript:

ADEV	Allan Deviation
AGC	Amplitude Gain Control
ARW	Angle Random Walk
CVG	Coriolis Vibratory Gyroscopes
DUT	Devices Under Test
FRB	Force-to-Rebalance
MEMS	Microelectromechanical Systems
MTN	Mechanical-Thermal Noise
OL	Open-Loop
PLL	Phase-Locked Loop
PSD	Power Spectral Density
Q-factor	Quality factor
QCL	Quadrature Control Loop
QMG	Quad Mass Gyroscope
RCL	Rate Control Loop
RRW	Rate Random Walk
SF	Scale-factor
TED	Thermoelastic Damping
ZRO	Zero Rate Output
